# Effect of Team-Based Care on Chronic Disease Control: Hypertension and Diabetes

**DOI:** 10.7759/cureus.86971

**Published:** 2025-06-29

**Authors:** Zainb A Alshaiti, Yasamiyan A Alburayh, Montaser A Bu-Khamseen, Qasem Al Jabr, Abdul Sattar Khan, Fatimah N Al Jamaan

**Affiliations:** 1 Familly Medicine, King Faisal University, Al-Ahsa, SAU; 2 Family Medicine, Al-Ahsa Family Medicine Academy, Al-Ahsa, SAU; 3 Family Medicine, American Board of Family Medicine, American Board of Geriatric Medicine, Al-Ahsa, SAU; 4 Family and Community Medicine, King Faisal University, Al-Ahsa, SAU

**Keywords:** diabetes mellitus, hypertension, management, saudi arabia, team-based care

## Abstract

Introduction: Chronic diseases are a major challenge for healthcare systems. Primary care providers face time constraints, leaving urgent care needs unmet. Team-based care (TBC) has emerged as an effective approach, fostering collaboration among healthcare professionals to improve patient outcomes and disease management.

Aim: The aim of this study was to investigate the impact of TBC on chronic disease management in Al-Ahsa, Saudi Arabia, with a focus on hypertension and diabetes control.

Methods: A retrospective study was conducted in all primary healthcare centers (PHCs) in Al-Ahsa, Saudi Arabia, from 2021 to 2025. The study included 23,495 patients, including 13,492 diabetic and 10,003 hypertensive patients. Data were collected from electronic health records. Statistical analysis was conducted using IBM SPSS Statistics for Windows, version 27, and paired sample t-tests were used to assess the effectiveness of TBC. The significance level for all statistical tests was set at p < 0.05.

Results: The study included 23,492 patients, including 13,492 diabetic and 10,003 hypertensive patients. TBC intervention significantly improved diabetic patients' glycemic control, evidenced by a mean HbA1c reduction from 7.5% to 7.2% across all demographic subgroups. Furthermore, TBC led to a statistically significant reduction in systolic blood pressure (SBP) among hypertensive patients across most demographic groups, with an overall mean decrease from 134.3 to 131.2 mmHg.

Conclusion: The study confirms the effectiveness of TBC in managing hypertension and diabetes. To maximize benefits, tailored interventions, regional adaptations, and comprehensive training for healthcare providers are crucial.

## Introduction

Chronic diseases pose significant challenges to healthcare systems and are considered to be the leading cause of death worldwide [1]. In 2021, the World Health Organization (WHO) reported that noncommunicable diseases accounted for 68% of the top 10 leading causes of death globally, accounting for seven out of 10 leading causes [2]. 

Global health faces a critical challenge from the escalating prevalence of hypertension and diabetes, with both conditions disproportionately affecting low- and middle-income countries (LMICs). Hypertension cases doubled from 650 million in 1990 to 1.3 billion by 2019, with nearly half of those affected unaware of their status. Similarly, diabetes prevalence surged from 200 million in 1990 to 830 million in 2022, contributing to over two million deaths in 2021 [3,4]. The literature showed that the prevalence of HTN in Saudi Arabia is 9.2%, with higher rates in women with significant regional variations [5]. Similarly, according to the International Diabetes Federation (IDF) in 2024, the prevalence of diabetes mellitus (DM) in Saudi Arabia is around 23.7% among adults [6]. 

The rising prevalence of these conditions necessitates innovative approaches to improve patient outcomes and control disease progression. It has been proven on many occasions that teams with diverse expertise are more adept at delivering comprehensive and well-coordinated healthcare for individuals dealing with intricate chronic illnesses [7]. Team-based care (TBC) is a promising strategy that combines healthcare professionals from different disciplines to manage chronic diseases. This approach involves physicians, nurses, pharmacists, dietitians, and behavioral health specialists, each with unique skills and expertise. The effectiveness of TBC relies on the integration of these professionals to address the comprehensive needs of patients with chronic diseases [8,9]. The literature revealed that the impact of TBC on hypertension control consistently demonstrates positive outcomes [10,11]. Collaborative efforts between physicians and pharmacists in medication management, patient education, and lifestyle interventions have shown improvements in blood pressure control [10].

TBC has proven effective in diabetes management by emphasizing patient education, medication adherence, and lifestyle modifications. Interventions involving multidisciplinary teams, including endocrinologists, nurses, dietitians, and pharmacists, have shown enhanced glycemic control and reduced complications [10,12,13]. As an integral component of Saudi Arabia's Vision 2030, the Health Sector Transformation Program (HSTP) is in the process of reshaping the health sector into a comprehensive, efficient, and integrated system that prioritizes the health of the population [14]. A recent study evaluating the impact of TBC on Saudi Arabia’s family medicine health provider attitude has shown that the residents generally displayed a positive attitude, particularly regarding the value of teamwork. However, there is a need for enhancement in their comprehension of the shared role of physicians within the team, which can be achieved through training and exposure to role models [15].

The increasing prevalence of chronic diseases like hypertension and diabetes in Saudi Arabia is a significant public health challenge. Current management strategies often fail to achieve optimal disease control, leading to increased morbidity, mortality, and healthcare costs. The study aims to provide evidence-based recommendations for optimizing chronic disease management strategies, contributing to better health outcomes and a more sustainable healthcare system. The findings can inform policy decisions, guide resource allocation, and improve clinical practice, ultimately leading to a healthier and more productive Saudi population. The overall goal of this study is to investigate the effect of TBC on the control of chronic diseases in Saudi Arabia including hypertension and diabetes. 

## Materials and methods

Study design and setting

This was a retrospective comparative study conducted in all primary healthcare centers (PHCs) in Al-Ahsa, Eastern Province, Saudi Arabia. The study compared data from two distinct periods: before TBC (January to November 2021) and after TBC (December 2021 to February 2025). The study received approval from the institutional review board (IRB) of PSBJH (approval no. H-05-HS-135), while formal consent was not taken due to the retrospective study design. The Saudi Ministry of Health (MOH) began implementing the TBC model in all regions/clusters in 2021, as part of Saudi Vision 2030. The TBC model aims to transition the delivery of primary healthcare services from solo practice to TBC. Teams consist of family physicians, nurses, health coaches, and case coordinators, with physicians providing physical examinations, nurses providing screening services, health coaches providing individualized health coaching, and case coordinators arranging referrals and appointment scheduling.

Population and sample size

The study included hypertensive or diabetic patients who presented to PHCs in Al-Ahsa City. A total of 23,492 patients (13,492 diabetic and 10,003 hypertensive) who met the inclusion criteria were included in the study. The criteria include Saudi males or females registered in the PHCs, Al-Ahsa, and diagnosed with diabetes mellitus (DM) or hypertension (HTN) or both. Patients who are not Saudi, do not follow up in one of the PHCs, are younger than 18 years old, or passed away before the application of the TBC system were excluded from the study. 

Data collection

Data were collected retrospectively from electronic health records using a standardized data collection form. The collected information included patient demographics (age, gender, marital status, and region) and, for diabetic patients (type 1 and type 2), HbA1c levels before and after the implementation of TBC. For hypertensive patients, systolic blood pressure (SBP) and diastolic blood pressure (DBP) measurements were collected before and after TBC implementation.

Data analysis

The statistical analysis was conducted using IBM SPSS Statistics for Windows, Version 27.0 (released 2019, IBM Corp., Armonk, NY). Descriptive statistics, including frequencies, percentages, means, and standard deviations, were used to summarize the sociodemographic profiles of the study participants. To assess the effectiveness of TBC, paired sample t-tests were utilized to compare the mean levels of HbA1c, SBP, and DBP before and after the intervention, respectively. The significance level for all statistical tests was set at p < 0.05.

## Results

Table 1 presents the sociodemographic characteristics of the study population, comprising 23,495 individuals with chronic diseases. Geographically, the eastern region accounted for the most significant proportion of the study population, with 7,303 individuals, representing 31.1% of the total. Within the disease-specific groups, the eastern region also exhibited the highest numbers, with 3,967 DM cases (29.4%) and 3,336 HTN cases (33.3%). The southern region had the smallest representation, with 2,890 individuals (12.3%) overall. Age distribution revealed that the 50-59 age group constituted the largest segment of the population, with 7,781 individuals (33.1%), and also had the highest numbers for DM (4,508, 33.4%) and HTN (3,273, 32.7%). The mean ages for the total population and the disease-specific groups were 55.8 ± 11.5, 55.7 ± 11.0 and 56.1 ± 12.1 years for total, DM, and HTN, respectively. Regarding gender, females comprised the majority of the study population, with 13,534 individuals (57.6%), and were also predominant in all disease categories: DM (8,199, 60.8%) and HTN (5,335, 53.3%). Marital status showed that the largest category was "Unknown" with 11,180 people (47.6%) of the total population and the largest within the disease groups. However, the "Married" category had the highest number when looking at the known marital status, with 10,523 individuals (44.8%) overall and also the highest number for DM (6,223, 46.1%) and HTN (4,300, 43.0%)

**Table 1:** Sociodemographic characteristics of study cases with chronic diseases (diabetes mellitus (DM), hypertension (HTN), and body mass index (BMI))

Sociodemographic		Total (N = 23,495)	DM (N = 13,492)	Hypertension (N = 10,003)
		No. (%)	No. (%)	No. (%)
Region	Eastern	7,303 (31.1%)	3,967 (29.4%)	3,336 (33.3%)
	Middle	6,976 (29.7%)	4,038 (29.9%)	2,938 (29.4%)
	Northern	5,449 (23.2%)	2,941 (21.8%)	2,508 (25.1%)
	Not reported	877 (3.7%)	877 (6.5%)	0 (0.0%)
	Southern	2,890 (12.3%)	1,669 (12.4%)	1,221 (12.2%)
Age in years	18-39	1,844 (7.8%)	1,005 (7.4%)	839 (8.4%)
	40-49	5,050 (21.5%)	3,012 (22.3%)	2,038 (20.4%)
	50-59	7,781 (33.1%)	4,508 (33.4%)	3,273 (32.7%)
	60-69	6,246 (26.6%)	3,598 (26.7%)	2,648 (26.5%)
	70+	2,574 (11.0%)	1,369 (10.1%)	1,205 (12.0%)
	Mean ± SD	55.8 ±11.5	55.7 ± 11.0	56.1 ± 12.1
Gender	Male	9,961 (42.4%)	5,293 (39.2%)	4,668 (46.7%)
	Female	13,534 (57.6%)	8,199 (60.8%)	5,335 (53.3%)
Marital status	Single	827 (3.5%)	457 (3.4%)	370 (3.7%)
	Married	10,523 (44.8%)	6,223 (46.1%)	4,300 (43.0%)
	Divorced / widow	965 (4.1%)	594 (4.4%)	371 (3.7%)
	Unknown	1,1180 (47.6%)	6,218 (46.1%)	4,962 (49.6%)

Table 2 illustrates the changes in HbA1c levels before and after TBC among 13,492 diabetic patients, categorized by demographic factors. The data reveal a consistent and statistically significant (p < 0.001) reduction in the mean HbA1c levels across all demographic subgroups. Regionally, regardless of location, patients experienced a decrease in HbA1c, with mean pre-TBC levels ranging from 7.4% to 7.6% and post-TBC levels ranging from 7.2% to 7.3%. Age stratification showed that all age groups benefited from TBC, with the 18-39 group demonstrating the most substantial reduction, from 7.1% to 6.7%. Both male and female patients showed significant improvements, with males experiencing a decrease from 7.7% to 7.3% and females from 7.4% to 7.2%. Similarly, all marital status categories, including "Unknown," showed significant reductions in HbA1c. The overall mean HbA1c decreased from 7.5% to 7.2%, with the range narrowing from 2.4-20.0% to 3.4-16.6% post-TBC.

**Table 2:** Changes in HbA1c before and after TBC by demographic factors among diabetic patients (N = 13,492) P: paired sample t-test, * P < 0.05 (significant), %: percentage. HbA1c: hemoglobin A1C, TBC: team-based care

Demographic		HbA1c before TBC	HbA1c after TBC	p-value
		(Mean ± SD)	(Mean ± SD)	
Region	Eastern	7.6 ± 2.0%	7.3 ± 1.8%	0.001*
	Middle	7.4 ± 2.0%	7.2 ± 1.8%	0.001*
	Northern	7.5 ± 2.0%	7.3 ± 1.8%	0.001*
	Not reported	7.6 ± 1.9%	7.2 ± 1.7%	0.001*
	Southern	7.5 ± 1.9%	7.2 ± 1.8%	0.001*
Age in years	18-39	7.1 ± 2.1%	6.7 ± 1.8%	0.001*
	40-49	7.2 ± 2.0%	7.0 ± 1.8%	0.001*
	50-59	7.6 ± 2.0%	7.3 ± 1.8%	0.001*
	60-69	7.7 ± 1.9%	7.5 ± 1.7%	0.001*
	70+	7.5 ± 1.7%	7.3 ± 1.6%	0.001*
Gender	Male	7.7 ± 2.0%	7.3 ± 1.8%	0.001*
	Female	7.4 ± 2.0%	7.2 ± 1.8%	0.001*
Marital status	Single	7.5 ± 2.1%	7.2 ± 1.9%	0.001*
	Married	7.5 ± 2.0%	7.3 ± 1.8%	0.001*
	Divorced / widow	7.7 ± 2.0%	7.4 ± 1.9%	0.001*
	Unknown	7.5 ± 2.0%	7.2 ± 1.8%	0.001*
Overall	Range	2.4 - 20.0%	3.4 - 16.6%	0.001*
	Mean SD	7.5 ± 2.0%	7.2 ± 1.8%	

Table 3 shows the changes in SBP before and after TBC among 10,003 hypertensive patients by demographic factors. The data demonstrate a statistically significant reduction in the mean SBP across most demographic groups (p < 0.05). Regionally, all areas showed significant decreases, with mean pre-TBC SBP ranging from 133.8 to 135.2 mmHg and post-TBC SBP ranging from 131.0 to 131.9 mmHg. Age-wise, all age groups experienced significant reductions, with the 70+ group showing the largest decrease from 137.5 to 131.9 mmHg. Both male and female patients exhibited significant reductions in SBP, with males decreasing from 134.4 to 131.3 mmHg and females from 134.2 to 131.2 mmHg. Among marital statuses, married, divorced/widowed, and unknown categories showed significant reductions. However, the single group did not demonstrate a statistically significant change (p = 0.235). The overall mean SBP decreased from 134.3 to 131.2 mmHg, with the range narrowing from 61-225 mmHg to 77-156 mmHg post-TBC.

**Table 3:** Changes in systolic blood pressure (SBP) before and after team-based care (TBC) by demographic factors among hypertension cases (N = 10,003) P: Paired sample t-test, * P < 0.05 (significant), mmHg: millimeters of mercury

Demographics		SBP before TBC	SBP after TBC	p-value
		Mean ± SD	Mean ± SD	
Region	Eastern	134.2 ± 17.1 mmHg	131.0 ± 11.4 mmHg	0.001*
	Middle	133.8 ± 16.9 mmHg	131.1 ± 11.6 mmHg	0.001*
	Northern	134.5 ± 16.7 mmHg	131.5 ± 11.0 mmHg	0.001*
	Southern	135.2 ± 18.3 mmHg	131.9 ± 11.2 mmHg	0.001*
Age in years	18-39	130.2 ± 16.1 mmHg	128.6 ± 10.9 mmHg	0.012*
	40-49	133.4 ± 16.0 mmHg	130.5 ± 10.9 mmHg	0.001*
	50-59	133.7 ± 17.0 mmHg	130.4 ± 11.0 mmHg	0.001*
	60-69	135.6 ± 17.3 mmHg	132.3 ± 11.3 mmHg	0.001*
	70+	137.5 ± 19.1 mmHg	131.9 ± 12.0 mmHg	0.001*
Gender	Male	134.4 ± 16.6 mmHg	131.3 ± 11.3 mmHg	0.001*
	Female	134.2 ± 17.5 mmHg	131.2 ± 11.4 mmHg	0.001*
Marital status	Single	131.7 ± 15.6 mmHg	130.6 ± 10.9 mmHg	0.235
	Married	134.7 ± 17.0 mmHg	130.7 ± 11.5 mmHg	0.001*
	Divorced / widow	136.4 ± 19.1 mmHg	132.6 ± 10.2 mmHg	0.001*
	Unknown	134.4 ± 17.2 mmHg	131.6 ± 11.3 mmHg	0.001*
Overall	Range	61-225 mmHg	77-156 mmHg	0.001*
	Mean ± SD	134.3 ± 17.1 mmHg	131.2 ± 11.3 mmHg	

Table 4 illustrates the changes in DBP before and after TBC among 10,003 hypertensive patients by demographic factors. The data reveal varying results regarding the effectiveness of TBC in reducing DBP. Regionally, the eastern, middle, and northern regions showed statistically significant (p < 0.05) reductions in DBP, with mean pre-TBC values around 74 mmHg decreasing slightly post-TBC. However, the southern region showed no significant change (p = 0.128). Age-wise, only the 60-69 and 70+ age groups demonstrated significant reductions in DBP, while the younger age groups showed no significant changes. Gender analysis revealed that only females experienced a significant reduction in DBP (p < 0.001), decreasing from 72.6 to 72.0 mmHg, whereas males showed no significant change. Among marital statuses, married and divorced/widowed categories exhibited significant reductions, but single and unknown categories did not. The overall mean DBP decreased significantly from 74.1 to 73.6 mmHg (p < 0.001), with the range narrowing from 70-142 mmHg to 46-113 mmHg post-TBC.

**Table 4:** Changes in diastolic blood pressure (DBP) before and after team-based care (TBC) by demographic factors among hypertension cases (N = 10,003) P: Paired sample t-test, * P < 0.05 (significant), mmHg: millimeters of mercury

Demographic		DBP before TBC	DBP after TBC	p-value
		(Mean ± SD)	(Mean ± SD)	
Region	Eastern	74.1 ± 10.4 mmHg	73.5 ± 9.6 mmHg	0.010*
	Middle	74.1 ± 10.6 mmHg	73.5 ± 9.8 mmHg	0.009*
	Northern	73.9 ± 10.7 mmHg	73.3 ± 10.2 mmHg	0.015*
	Southern	74.4 ± 10.9 mmHg	74.9 ± 9.7 mmHg	0.128
Age in Years	18-39	76.0 ± 11.3 mmHg	76.5 ± 10.1 mmHg	0.251
	40-49	76.4 ± 10.8 mmHg	76.6 ± 9.7 mmHg	0.567
	50-59	74.4 ± 10.4 mmHg	74.0 ± 9.8 mmHg	0.215
	60-69	72.4 ± 10.7 mmHg	71.5 ± 9.9 mmHg	0.001*
	70+	71.4 ± 9.9 mmHg	70.2 ± 9.7 mmHg	0.002*
Gender	Male	75.7 ± 10.7 mmHg	75.5 ± 9.6 mmHg	0.180
	Female	72.6 ± 10.3 mmHg	72.0 ± 9.8 mmHg	0.001*
Marital Status	Single	74.6 ± 11.4 mmHg	74.1 ± 9.7 mmHg	0.438
	Married	74.1 ± 10.5 mmHg	73.5 ± 9.8 mmHg	0.008*
	Divorced / Widow	72.8 ± 9.8 mmHg	71.3 ± 9.6 mmHg	0.015*
	Unknown	74.1 ± 10.7 mmHg	73.8 ± 9.9 mmHg	0.079
Overall	Range	70-142 mmHg	46-113 mmHg	0.001*
	Mean ± SD	74.1 ± 10.6 mmHg	73.6 ± 9.9 mmHg	

## Discussion

Among diabetic patients, this study proved the effectiveness of TBC in improving chronic disease management, particularly diabetes, as evidenced by significant HbA1c reductions across diverse demographic subgroups. These findings are consistent with real-world evidence from the Community Health Center (CHC) Initiative [9] and studies by Shojania et al. [11], which emphasize the value of multidisciplinary collaboration in enhancing glycemic control. The pronounced improvements in younger patients (18-39 years) highlight the importance of personalized, supportive care, consistent with research by Stellefson et al. [16], while older adults also benefited, suggesting TBC's adaptability to varying patient complexities. 

The variability of TBC across regions, including resource-limited settings, is further supported by studies like the Hypertension and Diabetes Care Project [17]. However, contextual adaptations, such as integrating community health workers and telehealth services in rural areas, as noted by Nutting et al. [18], are crucial for successful implementation. While this study's results are promising, they contrast with some meta-analyses, such as that by Zwar et al. [19], which reported modest improvements, emphasizing the need for standardized protocols and adequate training. In conclusion, this study contributes to the growing evidence supporting TBC as a transformative strategy for chronic disease management. Future research should focus on long-term sustainability, cost-effectiveness, and healthcare system integration to ensure widespread adoption and impact.

The effectiveness of TBC in improving chronic disease management, particularly for diabetes and hypertension, is evident in both Saudi Arabia and internationally, though with contextual variations. In Saudi Arabia, TBC models involving multidisciplinary teams have demonstrated significant improvements in glycemic control and blood pressure management in primary care settings [20-22]. These findings are consistent with international evidence, such as the CHC Initiative in the U.S., which also reported positive outcomes through multidisciplinary care [9]. However, the implementation of TBC in Saudi Arabia is influenced by unique challenges, including cultural barriers, limited patient awareness, and regional healthcare infrastructure disparities, as well as downsides to TBC, for example, challenges with communication and coordination, and more appointments for the patients. International studies, particularly in high-income countries, often benefit from more established healthcare systems and higher patient engagement [16]. For example, the Hypertension and Diabetes Care Project in low-income U.S. communities utilized community health workers and telehealth services to bridge care access gaps [4], while Australian rural TBC models incorporated remote monitoring and culturally tailored interventions for indigenous populations [19]. Despite these differences, the fundamental principles of TBC, i.e., collaboration, patient-centered care, and continuous support, remain universally applicable. In Saudi Arabia, scaling up TBC necessitates addressing systemic challenges such as workforce training and resource allocation while integrating international best practices to ensure equitable and sustainable implementation.

Moreover, our study demonstrates the effectiveness of TBC in improving blood pressure control among hypertensive patients, with significant reductions in SBP across most demographic groups. These results are consistent with national and international studies, strengthening the value of TBC in chronic disease management. In Saudi Arabia, the observed reduction in SBP from 134.3 mmHg to 131.2 mmHg post-TBC is consistent with findings from Alshowair A et al. (2023), who reported similar improvements in blood pressure control through multidisciplinary care models in PHCs [22]. The pronounced benefit in older adults (70+ years), with SBP decreasing from 137.5 mmHg to 131.9 mmHg, mirrors international evidence, such as the Sprint Trial, which highlighted the importance of intensive, team-based interventions in reducing cardiovascular risks in elderly populations [23].

However, the lack of significant SBP reduction in single patients and the limited impact on DBP in certain subgroups, such as males and younger age groups, suggests that TBC may need tailored adaptations to address specific patient needs. The absence of significant DBP changes in the southern region and among single patients may reflect cultural or socioeconomic factors influencing engagement with care. This contrasts with international studies, such as the Hypertension and Diabetes Care Project in the U.S., where TBC was effective across all demographics, likely due to the integration of community health workers and culturally sensitive interventions [17]. Similarly, a study in Australia found that TBC models incorporating telehealth and remote monitoring were particularly effective in rural and underserved populations, highlighting the importance of context-specific adaptations [24]. The significant reduction in DBP among females and older adults further underscores the potential of TBC to address gender- and age-specific health disparities. This aligns with findings from the Women’s Health Initiative, which demonstrated that collaborative care models significantly improved blood pressure control in women, particularly those with multiple comorbidities [25]. However, the limited impact on DBP in males and younger patients suggests that these groups may require more targeted interventions, such as lifestyle modification programs or digital health tools, to enhance engagement and outcomes.

Strengths and limitations of the study

The study, which included 23,495 patients, has strengths in its large sample size and use of electronic health records to provide reliable data on hypertension and diabetes. The results show significant improvements in hemoglobin A1C (HbA1c) and SBP, supporting the effectiveness of total blood circulation (TBC). However, limitations include the retrospective design, lack of a control group, limited detail on specific TBC interventions, and its focus on blood sugar and blood pressure, omitting other important patient outcomes. Health systems vary significantly across countries, and TBC may be more or less effective or applicable in specific regions.

## Conclusions

This study confirms the effectiveness of TBC in managing hypertension and diabetes, evidenced by significant reductions in SBP and HbA1c across most demographic groups. To maximize TBC's benefits, tailored interventions, such as gender- and age-specific approaches and regional adaptations using community health workers and telehealth, are crucial. Comprehensive training for healthcare providers in multidisciplinary care and patient-centered approaches is also essential.
